# Bibliometric Analysis of the 100 Most‐Cited Articles on the Methods of Shade‐Matching in Dentistry

**DOI:** 10.1002/cre2.70037

**Published:** 2024-11-03

**Authors:** Farah Rashid, James Dudley

**Affiliations:** ^1^ Adelaide Dental School The University of Adelaide South Australia Australia

**Keywords:** bibliometric analysis, colorimeter, dentistry, shade‐matching, spectrophotometer, visual method

## Abstract

**Objectives:**

The purpose of this bibliometric analysis is to identify the 100 most cited articles and delve into citation metrics to gain insights into the evolving trends in shade‐matching methods in dentistry.

**Material and Methods:**

Following PRISMA guidelines, two reviewers conducted a structured search in Scopus using keyword‐based search strings. The top 100 articles were selected based on predefined criteria, and their bibliometric data were extracted. *Harzing's Publish Or Perish* and *VOSviewer* were used to generate the bibliographic network.

**Results:**

Between 1989 and 2017, the top 100 articles were published and obtained citations ranging from 560 to 48. Twenty‐eight articles received over 100 citations, deemed classic. The *Journal of Prosthetic Dentistry* and the United States of America were the primary contributors. In‐vitro experimental studies employing restorative material samples were prevalent, with spectrophotometers being the favored method of color analysis. Using Python, Spearman's correlation coefficient resealed positive correlations between citation count and age of publication (*r* = 0.16, *p* = 0.12) and between citation count and the impact factor of the journal (*r* = 0.35, *p* < 0.05). However, a negative correlation was observed between citation density and age of publication (*r* = −0.46, *p* < 0.05).

**Conclusions:**

This study demonstrated a significant upward trajectory of citation count in shade‐matching in dentistry, reflecting high‐quality scientific contributions.

## 1 Introduction

Contemporary dentistry has moved beyond the traditional restoration of tooth function with a heightened focus on enhancing esthetic aspects, driven in part by the growing importance of social media and digital multimedia communications (Rashid, Farook, and Dudley 2023). Accurately matching the color of natural teeth is acknowledged as a crucial and challenging aspect of dentistry and is influenced by an individual's perceptibility and acceptability threshold toward the color, with metamerism adding further complexity (Rashid, Farook, and Dudley 2023; Joiner 2004; Paul et al. 2002).

The recognition of shade‐matching challenges has fueled a growing demand for scientific knowledge pertaining to clinical procedures and information on equipment capable of effectively conducting tooth shade analysis. Some studies have delved into the theories of color and color spaces (Joiner 2004) others explored the optical properties of teeth to simplify the shade‐matching procedure (Ten Bosch and Coops 1995; Douglas 1997), and some detailed thorough procedures for measuring tooth color using various methods such as visual, spectrophotometric, colorimetric, and photographic modalities (Eimar et al. 2011; Shokry et al. 2006; Hammad 2003; Cal et al. 2004). Further studies compared different methods to establish a standard procedure that can determine the tooth color perfectly (Yılmaz et al. 2008; Braun, Jepsen, and Krause 2007; Guan et al. 2005), while others discussed external factors (lighting and environmental variations) that may influence or hinder the accuracy of color analysis (Rashid et al. 2022).

Research has explored the topic of tooth shade‐matching in dentistry; however, identifying outstanding research from the vast volume of publications has been challenging (Praveen et al. 2020). To aid in identifying crucial research areas and track their growth and development, a quantitative statistical and mathematical approach was introduced termed “bibliometric analysis” (Alhajj et al. 2023; Ahmad et al. 2020). Unlike academic research and review papers that delve into specific topics, bibliometric analysis is centered on comprehending global research trends within a particular field by analyzing the bibliometric outputs of academic publications, utilizing databases such as Scopus or Web of Science (WoS) (Alsharif, Salleh, and Baharun 2020). In recent years, there has been a growing inclination in health and medical sciences toward citation analysis, which stands out as the most employed bibliometric analysis tool (Ahmad, Della Bella, and Stoddart 2020). The presence of a high citation index in a study does not necessarily indicate the quality of the research; instead, it highlights the connections between published papers that share similar topics within a scholarly landscape (Praveen et al. 2020; McBurney and Novak 2002). In dentistry, bibliometric analyses have been conducted in various specialized fields, such as periodontology, prosthodontics, orthodontics, pediatrics, implantology, oral and maxillofacial surgery, and endodontics. Currently, there is a notable absence of bibliometric analyses exclusively focused on the methods of shade‐matching, particularly, in the context of shade‐matching of tooth or restorative materials in dentistry.

The aim of this bibliometric analysis was to identify and analyze the characteristics of the 100 most cited articles on the methods of shade‐matching in dentistry. The characteristic evaluation included analyzing the article's citation metrics, year of publication, influential journals and authors, keywords, and evolving different techniques of shade‐matching that contribute to shaping research in dentistry's color sciences. Additionally, this study aimed to investigate the interrelationships among contributing factors such as citation metrics, the impact factor (IF) of a journal, and the age of the publications that may influence the shifting trends in shade‐matching methods in dentistry.

## 2 Material and Methods

### 2.1 Information Sources

Following the Preferred Reporting Items for Systematic Review and Meta‐Analysis (PRISMA) guidelines (Liberati 2009), two reviewers independently collected the data from Elsevier's Scopus, which was cross‐referenced with information from Google Scholar to ensure a comprehensive analysis. The title and abstract search were conducted between 1 February 2024 and 21 February 2024 by selecting “article title, abstract, keyword” within the Scopus database. Articles were sought in the subject category of “dentistry” starting from 1966 to 2023.

### 2.2 Eligibility Criteria for Bibliometric Analysis

This bibliometric analysis encompassed original scientific articles, in‐vivo and in‐vitro experimental studies, review articles, case reports, case series, clinical trials, conference papers, book, and book chapters that explicitly focused on different methods of shade‐matching on the tooth and restorative dental materials through color evaluation. Articles were identified using keyword‐based search strings in the database, and predefined inclusion and exclusion criteria were applied during the title and abstract screening process to select articles for further analysis.

The inclusion criteria were as follows: (1) articles with a minimum citation count (CC) of five; (2) articles that discussed different techniques (i.e., visual, instrumental: spectrophotometer, spectroradiometer, colorimeter, and photographs, or a combination of both visual and instrumental) of detecting color from the teeth or restorative material surfaces; and (3) articles that exclusively concentrated on tooth bleaching while integrating color measurement techniques were included; however, those solely discussing bleaching methods without addressing color parameters were excluded for further analysis.

The exclusion criteria applied to articles were as follows: (1) articles without shade‐matching analysis on human teeth samples, shade guides, and restorative dental materials; (2) non‐English articles lacking English translations; (3) studies that evaluated shade‐matching solely on functional orthodontic dental appliances, metal alloys, denture liners, and maxillofacial prosthetic materials; (4) studies that collected color values from the microstructural level; and (5) nonscientific literature, such as opinions, notes, editorials, surveys, and letters lacking empirical data.

### 2.3 Search Strategy

Customized keyword‐based search strings using Boolean Logic with wildcard characters (Rashid et al. 2020; Farook et al. 2023) were used to interrogate the Scopus database. The following search string was used: “tooth color” OR “tooth colour” OR “tooth shade*” OR “tooth shade‐matching” OR “tooth shade evaluation” OR “tooth color matching” OR “tooth colour matching” OR “teeth color” OR “teeth colour” OR “teeth shade*” OR “teeth shade‐matching” OR “teeth shade evaluation” OR “teeth color matching” OR “teeth colour matching” OR “tooth shade mismatch” OR “tooth color mismatch” OR “tooth colour mismatch” OR “teeth color mismatch” OR “teeth colour mismatch” OR “teeth shade mismatch” OR “visual tooth color” OR “visual teeth color” OR “tooth image* color” OR “tooth image* colour” OR “teeth image* color” OR “teeth image* colour” OR “spectrophotometer” OR “spectrophotometric” OR “colorimeter” OR “colourimeter” OR “colorimetric” OR “colourimetric” AND TITLE‐ABS‐KEY (“dent*” OR “dental” OR “dentistry”) AND TITLE‐ABS‐KEY (“analys?s” OR “analy?3” OR “tooth color analys?s technique*” OR “tooth colour analys?s technique*” OR “teeth color evolution” OR “teeth colour evaluation” OR “tooth color evaluation” OR “tooth colour evaluation” OR “teeth color analys?s technique*” OR “teeth colour analys?s technique*”)) AND (LIMIT‐TO (SUBJAREA, “dent”)).

### 2.4 Data Selection Process

Following customized keyword‐based search strings, a list of articles was retrieved from Scopus, and these articles were then organized in descending order based on their respective CCs. Adhering to predefined inclusion and exclusion criteria, two reviewers (F.R, J.D) independently conducted the title and abstract screening using Endnote (Version 20.1, UK) library. Any disagreements were resolved by performing further discussion. Among the selected papers, the 100 most cited papers were chosen for this bibliometric analysis and downloaded in the Research Information System (*RIS*) file format. To gather additional bibliometric data, the *RIS* file was imported into *Harzing's Publish Or Perish software* (Version 8.2.3944, UK) (Harzing). Subsequently, a Comma‐separated values (*CSV*) file containing the 100 selected most cited articles was downloaded for further data visualization purposes.

### 2.5 Data Items

The following key data were extracted from the selected articles: article title, authors name, citation metrics [CC, citation density (CD), current citation index (CCI) (citation received in 2023), years of citation], year of publication, journal name and its current IF from Journal Citation Report (JCR) by Clarivate, keywords, country of origin, study design (i.e., clinical and comparative observational study, experimental study, case–control, review articles, cross‐sectional study, clinical trials, and conference proceedings), nature of the study (i.e., in‐vivo, ex‐vivo, in‐vitro), study samples (i.e., restorative material, tooth, shade guide), and primary method of shade analysis. To assess the shade analysis methods from the tooth and restorative material surfaces, articles were further categorized into three groups: (1) visual, (2) instrumental (spectrophotometric, colorimetric, and photographic), and (3) a combination of both visual and instrumental approaches.

### 2.6 Statistical Analysis

An open‐source data visualization software named “Visualization of Similarities” (*VOSviewer*) (Version 1.6.20, Centre for Science and Technology Studies, Leiden University, the Netherlands) was utilized to generate maps to illustrate similarities among selected articles, with bubbles symbolizing keywords, country of origin, and author's contributions. Furthermore, a comprehensive correlation analysis was undertaken using an open‐source Python programming language. The codes were written and executed using “Google Colaboratory” known as Google Colab (Version 3.10, Google Research, CA) to explore potential relationships between CC—age of publication, CD—age of publication, CC—IF of the journal. The normality of data was assessed by using the Shapiro–Wilk test and Spearman's rank correlation coefficient was applied to evaluate the correlation coefficient.

## 3 Results

### 3.1 Literature Retrieval

**Figure 1:**
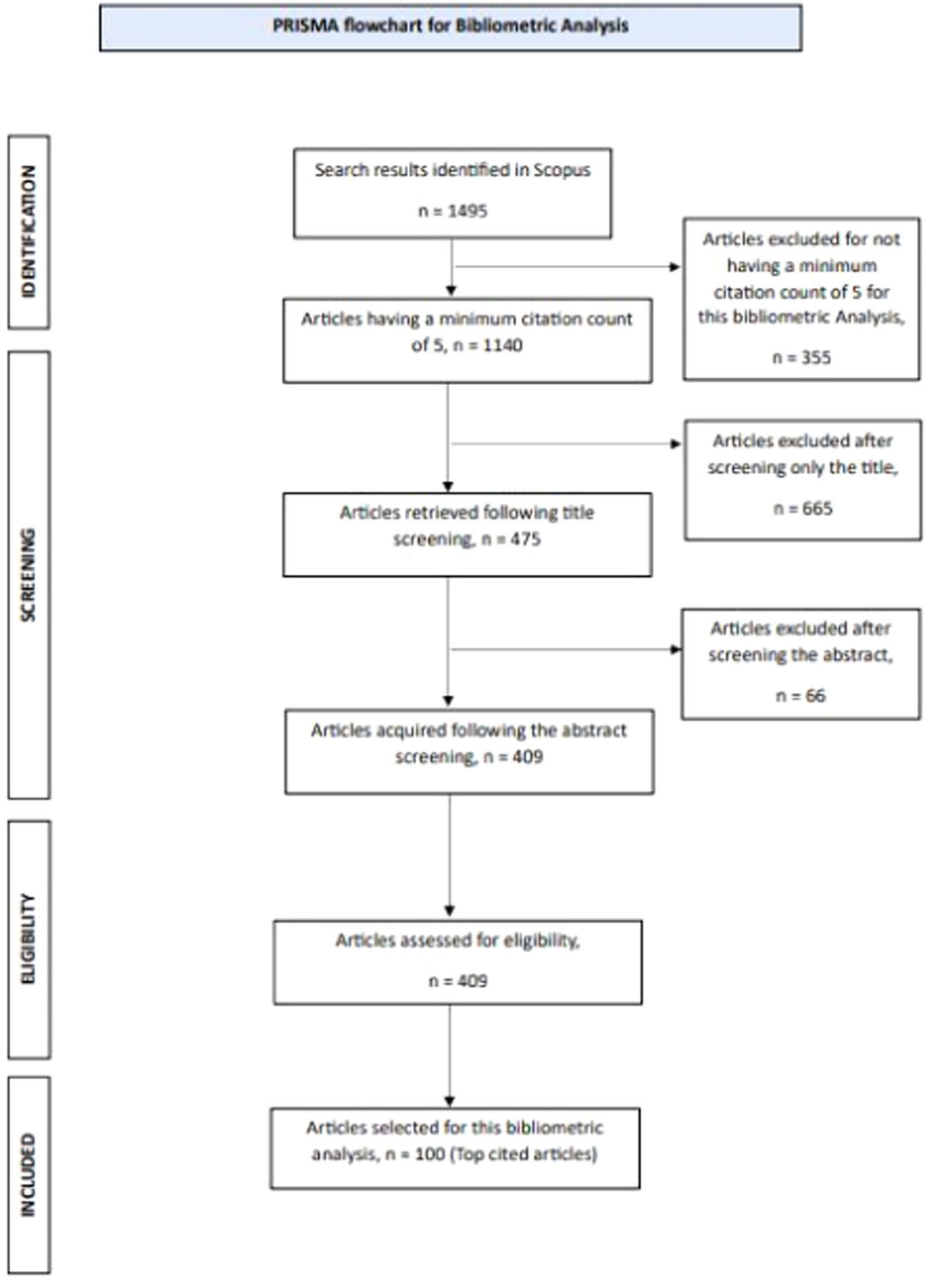
Preferred Reporting Items for Systematic Review and Meta‐Analysis (PRISMA) flowchart.

In Scopus, a total of 1495 articles were initially identified of which 1140 articles met the first inclusion criteria, which required a minimum CC of five. Following title screening, 665 articles were excluded, leaving 475 articles for further evaluation. Upon abstract screening, an additional 66 articles were excluded (Table S1). This resulted in 409 articles meeting the predefined inclusion criteria, from which only the top 100 most cited articles were selected for this bibliometric analysis (Figure 1).

### 3.2 Analysis of Citation Metrics

Table 1 shows the 100 most cited articles in the method of shade‐matching in dentistry, together with their citation metrics. These selected publications obtained a total of 10,341 citations, with individual CCs ranging from 48 to 560 and an average of 103.41 citations per article.

**Table 1:** Citation metrics of the 100 most cited articles in the methods of shade‐matching in dentistry.

Article Rank	Author name and year of publication	Title	Citation count	Citation density (citations per year	Current citation index	Age of publication
1.	Joiner 2004	Tooth color: A review of the literature	560	28	49	20
2.	Chu et al. 2010	Dental color matching instruments and systems. Review of clinical and research aspects	375	26.79	46	14
3.	Paul et al. 2002	Visual and spectrophotometric shade analysis of human teeth	362	16.45	25	22
4.	Seghi et al. 1989	Visual and Instrumental Colorimetric Assessments of Small Color Differences on Translucent Dental Porcelain	346	9.89	8	35
5.	Ertaş et al. 2006	Color stability of resin composites after immersion in different drinks	303	16.83	33	18
6.	Ten Bosch 1995	Tooth Color and Reflectance as Related to Light Scattering and Enamel Hardness	262	9.03	13	29
7.	Dietschi et al. 1994	Comparison of the color stability of ten new‐generation composites: An in vitro study	246	8.2	12	30
8.	Villalta et al. 2006	Effects of staining and bleaching on color change of dental composite resins	244	13.56	18	18
9.	Vichi et al. 2004	Color and opacity variations in three different resin‐based composite products after water aging	234	11.7	17	20
10.	Nasim et al. 2010	Color stability of microfilled, microhybrid, and nanocomposite resins ‐ An in vitro study	224	16	66	14
11.	Joiner 2017	Tooth color and whiteness: A review	156	22.29	44	7
12.	Seghi et al. 1989	Performance Assessment of Colorimetric Devices on Dental Porcelains	153	4.37	4	35
13.	O'Brien et al. 1990	A New, Small‐color‐difference Equation for Dental Shades	146	4.29	3	34
14.	Dozić et al. 2007	Performance of five commercially available tooth color‐measuring devices	141	8.29	7	17
15.	Gönülol. 2012	The effects of finishing and polishing techniques on surface roughness and color stability of nanocomposites	138	11.5	14	12
16.	Jarad et al. 2005	The use of digital imaging for color matching and communication in restorative dentistry	134	7.05	7	19
17.	Vallés et al. 2013	Influence of light and oxygen on the color stability of five calcium silicate‐based materials	134	12.18	10	11
18.	Hammad 2003	Intrarater repeatability of shade selections with two shade guides	132	6.29	6	21
19.	Joiner et al. 2008	A review of tooth color and whiteness	128	8	12	16
20.	Tung et al. 2002	The repeatability of an intraoral dental colorimeter	126	5.73	2	22
21.	Paul et al. 2004	Conventional visual vs spectrophotometric shade taking for porcelain‐fused‐to‐metal crowns: A clinical comparison	126	6.3	6	20
22.	Chaiyabutr et al. 2011	Effect of abutment tooth color, cement color, and ceramic thickness on the resulting optical color of a CAD/CAM glass‐ceramic lithium disilicate‐reinforced crown	124	9.54	12	13
23.	O'Brien et al. 1997	Color distribution of three regions of extracted human teeth	119	4.41	6	27
24.	Sarac et al. 2006	The effect of polishing techniques on the surface roughness and color change of composite resins	118	6.56	10	18
25.	Wee et al. 2002	Variation in color between intended matched shade and fabricated shade of dental porcelain	116	5.27	4	22
26.	Yannikakis et al. 1998	Color stability of provisional resin restorative materials	111	4.27	9	26
27.	Fondriest 2003	Shade matching in restorative dentistry: The science and strategies	107	5.1	5	21
28.	Turgut 2011	Color stability of laminate veneers: An in vitro study	102	7.85	7	13
29.	Douglas 2000	Color stability of new‐generation indirect resins for prosthodontic application	99	4.13	4	24
30.	Sarac et al. 2006	The effects of porcelain polishing systems on the color and surface texture of feldspathic porcelain	98	5.44	8	18
31.	Yuan et al. 2007	Defining a natural tooth color space based on a 3‐dimensional shade system	98	5.76	5	17
32.	Ozturk et al. 2008	The effect of ceramic thickness and number of firings on the color of two all‐ceramic systems	95	5.94	3	16
33.	Horn et al. 1998	Sphere spectrophotometer versus human evaluation of tooth shade	94	3.62	2	26
34.	Douglas 1997	Precision of in vivo colorimetric assessments of teeth	93	3.44	1	27
35.	Marconyak et al. 2016	A comparison of coronal tooth discoloration elicited by various endodontic reparative materials	93	11.63	11	8
36.	Lindsey 2007	Perceptibility and acceptability of CIELAB color differences in computer‐simulated teeth	92	5.41	11	17
37.	Kohli et al. 2015	Spectrophotometric analysis of coronal tooth discoloration induced by various bioceramic cements and other endodontic materials	92	10.22	10	9
38.	Buchalla et al. 2002	The effect of water storage and light exposure on the color and translucency of a hybrid and a microfilled composite	91	4.14	2	22
39.	Paravina 2009	Performance assessment of dental shade guides	91	6.07	8	15
40.	Catelan et al. 2011	Color stability of sealed composite resin restorative materials after ultraviolet artificial aging and immersion in staining solutions	91	7	6	13
41.	Shokry et al. 2006	Effect of core and veneer thicknesses on the color parameters of two all‐ceramic systems	88	4.89	6	18
42.	Kilinc et al. 2011	Resin cement color stability and its influence on the final shade of all‐ceramics	87	6.69	8	13
43.	De Oliveira et al. 2015	Effect of different photoinitiators and reducing agents on cure efficiency and color stability of resin‐based composites using different LED wavelengths	86	9.56	5	9
44.	Archegas et al. 2011	Color stability and opacity of resin cements and flowable composites for ceramic veneer luting after accelerated aging	84	6.46	5	13
45.	Shokouhinejad et al. 2016	Evaluation and Comparison of Occurrence of Tooth Discoloration after the Application of Various Calcium Silicate‐based Cements: An Ex Vivo Study	84	10.5	9	8
46.	Kim 2014	Effect of the number of coloring liquid applications on the optical properties of monolithic zirconia	83	8.3	9	10
47.	Seghi 1990	Effects of Instrument‐measuring Geometry on Colorimetric Assessments of Dental Porcelains	79	2.32	1	34
48.	Sham et al. 2004	Color stability of provisional prosthodontic materials	78	3.9	5	20
49.	Paravina et al. 2007	Optimization of tooth color and shade guide design: Clinical research	75	4.41	5	17
50.	Imirzalioglu et al. 2010	Color stability of denture acrylic resins and a soft lining material against tea, coffee, and nicotine	74	5.29	7	14
51.	Shulman et al. 2004	Perceptions of desirable tooth color among parents, dentists and children	73	3.65	3	20
52.	Kolbeck et al. 2006	Discoloration of facing and restorative composites by UV‐irradiation and staining food	72	4	6	18
53.	Falkensammer et al. 2013	Color stability of different composite resin materials	72	6.55	13	11
54.	Haselton et al. 2005	Color stability of provisional crown and fixed partial denture resins	71	3.74	5	19
55.	Gürdal et al. 2002	The effects of mouthrinses on microhardness and color stability of esthetic restorative materials	70	3.18	3	22
56.	Hosoya, 1999	Five‐year color changes of light‐cured resin composites: Influence of light‐curing times	68	2.72	3	25
57.	Yilmaz et al. 2008	Color stability of glazed and polished dental porcelains	68	4.25	6	16
58.	Heydecke et al. 2001	In vitro color stability of double‐layer veneers after accelerated aging	67	2.91	6	23
59.	Wee et al. 2007	Use of a porcelain color discrimination test to evaluate color difference formulas	67	3.94	5	17
60.	Furuse et al. 2008	Color‐stability and gloss‐retention of silorane and dimethacrylate composites with accelerated aging	67	4.19	3	16
61.	Braun et al. 2007	Spectrophotometric and visual evaluation of vital tooth bleaching employing different carbamide peroxide concentrations	66	3.88	0	17
62.	Arocha et al. 2014	Color stainability of indirect CAD‐CAM processed composites vs conventionally laboratory‐processed composites after immersion in staining solutions	66	6.6	10	10
63.	Cal et al. 2004	Application of a digital technique in evaluating the reliability of shade guides	64	3.2	4	20
64.	Kielbassa et al. 2009	In vitro comparison of visual and computer‐aided pre‐ and post‐tooth shade determination using various home bleaching procedures	64	4.27	3	15
65.	Li et al. 2009	Spectrophotometric evaluation of the optical influence of core build‐up composites on all‐ceramic materials	64	4.27	7	15
66.	Guan et al. 2005	The measurement of tooth whiteness by image analysis and spectrophotometry: A comparison	63	3.32	3	19
67.	Lim et al. 2010	Spectroradiometric and spectrophotometric translucency of ceramic materials	62	4.43	4	14
68.	Igiel et al. 2017	Reliability of visual and instrumental color matching	62	8.86	11	7
69.	Ma et al. 1997	Effects of chemical disinfectants on the surface characteristics and color of denture resins	61	2.26	3	27
70.	Azer et al. 2006	Effect of esthetic core shades on the final color of IPS Empress all‐ceramic crowns	61	3.39	1	18
71.	Noie et al. 1995	Color stability of resin cements after accelerated aging	60	2.07	5	29
72.	Lee et al. 2007	Layered color of all‐ceramic core and veneer ceramics	60	3.53	1	17
73.	Coşkun Akar et al. 2014	Effects of surface‐finishing protocols on the roughness, color change, and translucency of different ceramic systems	60	6	4	10
74.	Ragain. 2001	Minimum color differences for discriminating mismatch between composite and tooth color	58	2.52	0	23
75.	Sa Y. et al. 2012	Effects of two in‐office bleaching agents with different pH values on enamel surface structure and color: An in situ versus in vitro study	58	4.83	5	12
76.	Donahu et al. 1991	Shade color discrimination by men and women	57	1.73	2	33
77.	Browning 2003	Use of shade guides for color measurement in tooth‐bleaching studies	57	2.71	2	21
78.	Chu 2003	Use of a reflectance spectrophotometer in evaluating shade change resulting from tooth‐whitening products	57	2.71	1	21
79.	Brook et al. 2007	The clinical measurement of tooth color and stain	57	3.35	0	17
80.	Paravina et al. 2007	New shade guide for evaluation of tooth whitening ‐ Colorimetric study	57	3.35	1	17
81.	Fay et al. 1999	Color of restorative materials after staining and bleaching	56	2.24	1	25
82.	Azer et al. 2011	Effect of substrate shades on the color of ceramic laminate veneers	56	4.31	7	13
83.	Domingos et al. 2011	Composite resin color stability: Influence of light sources and immersion media	55	4.23	6	13
84.	Keskin et al. 2015	Color stabilities of calcium silicate‐based materials in contact with different irrigation solutions	55	6.11	3	9
85.	Ishikawa‐Nagai et al. 2005	Reproducibility of tooth color gradation using a computer color‐matching technique applied to ceramic restorations	54	2.84	2	19
86.	Kimet al. 2013	Effect of polishing and glazing on the color and spectral distribution of monolithic zirconia	54	4.91	7	11
87.	Rosenstiel et al. 1991	Duration of tooth color change after bleaching	51	1.55	1	33
88.	Uludag et al. 2007	The effect of ceramic thickness and number of firings on the color of ceramic systems: An in vitro study	51	3	2	17
89.	Yu B. 2008	Influence of color parameters of resin composites on their translucency	51	3.19	3	16
90.	Gehrkea et al. 2009	Comparison of in vivo visual, spectrophoto‐metric and colorimetric shade determination of teeth and implant‐supported crowns	50	3.33	5	15
91.	Li Q. et al. 2009	Color and surface analysis of carbamide peroxide bleaching effects on the dental restorative materials in situ	50	3.33	4	15
92.	Doray et al. 1997	Accelerated aging affects the color stability of provisional restorative materials	49	1.81	1	27
93.	Barrett et al. 2002	Influence of tab and disk design on shade matching of dental porcelain	49	2.23	3	22
94.	Derdilopoulou et al. 2007	Evaluation of visual and spectrophotometric shade analyses: A clinical comparison of 3,758 teeth	49	2.88	3	17
95.	Judeh, 2009	A comparison between conventional visual and spectrophotometric methods for shade selection	49	3.27	4	15
96.	Sabatini et al. 2012	Color stability of ten resin‐based restorative materials	49	4.08	5	12
97.	Mutlu‐Sagesen et al. 2001	Color stability of different denture teeth materials: an in vitro study	48	2.09	5	23
98.	Karamouzos et al. 2010	Tooth‐color assessment after orthodontic treatment: A prospective clinical trial	48	3.43	4	14
99.	Eimar et al. 2011	The role of enamel crystallography on tooth shade	48	3.69	3	13
100.	Lasserre et al. 2011	A comparison between a new visual method of color matching by intraoral camera and conventional visual and spectrometric methods	48	3.69	3	13

Based on CC, an article titled “Tooth color: A review of the literature” authored by Jonier stood as the top‐ranked article (CC = 560, CD = 28) over its 20‐year publication period since its release in the *Journal of Dentistry* in 2004. Following closely was a conference proceeding, titled “Dental color matching instruments and systems. Review of clinical and research aspects,” published by Chu et al. in the *Journal of Dentistry* in 2010 (CC = 375, CD = 26.79) during its 14‐year publication span. The third most cited article, “Visual and spectrophotometric shade analysis of human teeth” by Paul et al. published in the *Journal of Dental Research* in 2002 (CC = 362, CD = 16.45) over its 22‐year publication period. Using the CCI in 2023, a conference proceeding by Nasim et al. published in the *Journal of Dentistry* claimed the top position with a newly added CC of 66. Joiner and Chu et al. secured the second and third positions, respectively, with CCI of 49 and 46, respectively. Additionally, another article by Joiner et al. published in the *Journal of Dentistry* in 2017 ranked fourth due to receiving 44 new citations in 2023. In terms of CD, Joiner's articles in 2004 and 2017, along with Chu et al.'s article in 2010, achieved the highest scores of 28, 22.29, and 26.79, respectively, securing the top three positions.

**Figure 2:**
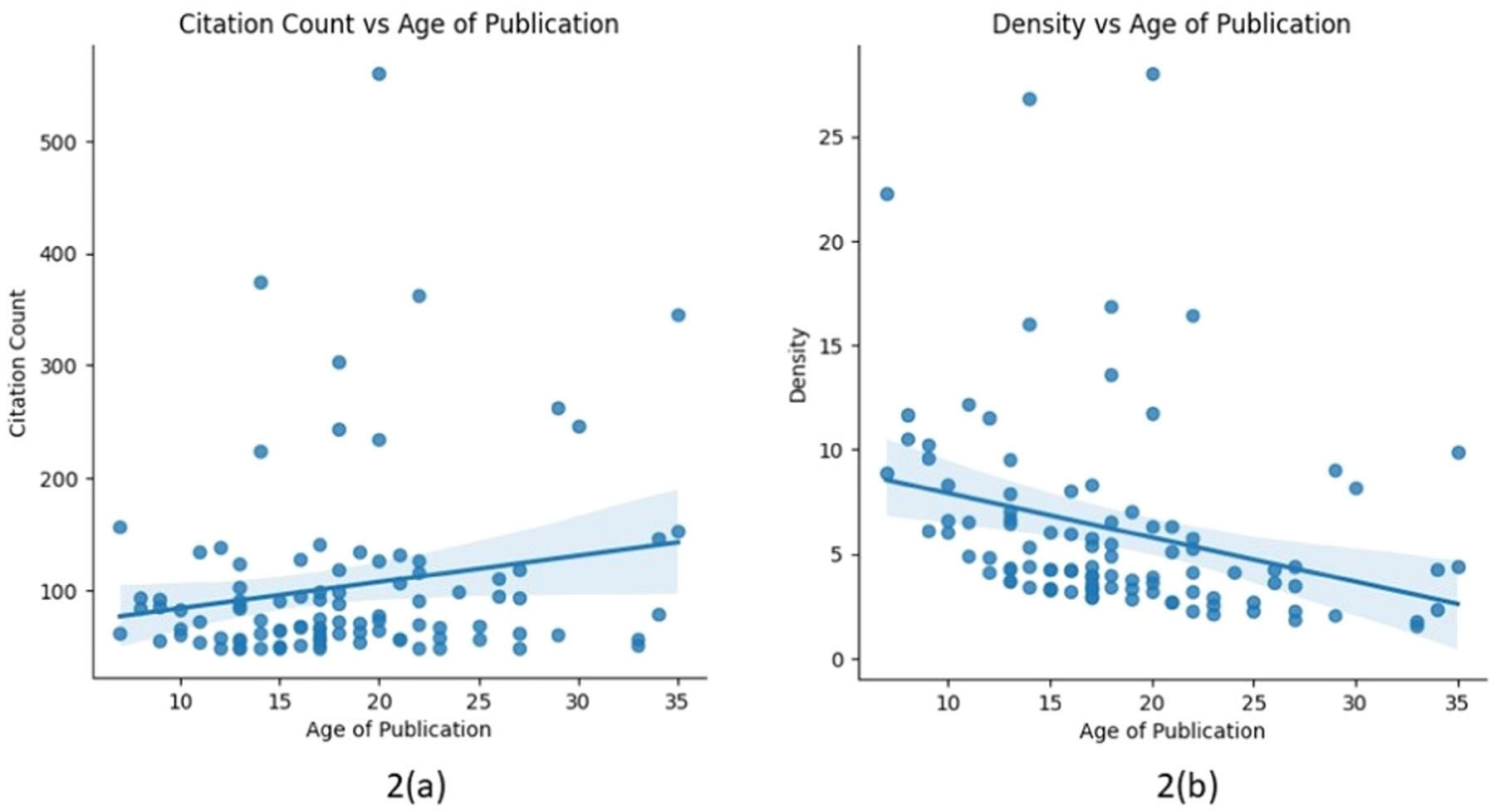
(a) Correlation analysis between citation count and age of publication and (b) Correlation analysis between citation density and age of publication.

According to the Shapiro–Wilk test the data for the CC, CD, and age of publication was not normally distributed (*p* < 0.05). When Spearman's correlation coefficient was applied to the data to evaluate the relationship between CC and age of publication, the result suggested a weak positive correlation trend (*r* = 0.16, *p* = 0.12) between the variables (Figure 2a). While this correlation was not deemed statistically significant, it suggested a subtle possibility that newer articles receive slightly more citations compared to older articles. Moreover, the correlation analysis between CD and age of publication yielded a statistically significant negative correlation coefficient (*r* = −0.46, *p* < 0.05), indicating that as the age of publication increases the CD decreases. Thus, the older articles tended to have lower CD compared to newer articles (Figure 2b). A Python code script detailing the conducted statistical analysis has been included in Table S2 for reference.

### 3.3 Top Publication Year

**Figure 3:**
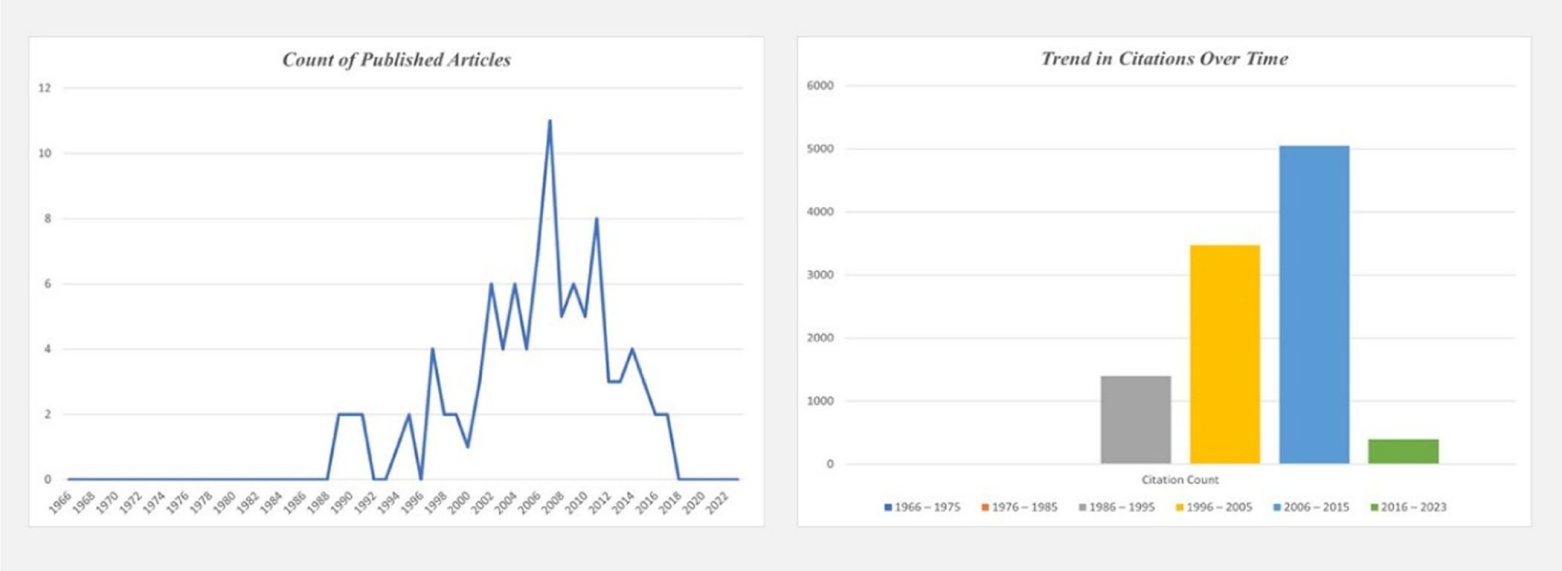
Citation trends on shade‐matching studies over the years.

The top 100 most cited articles on the methods of shade‐matching in dentistry were published between 1989 and 2017. The majority of these highly cited articles were published from 2006 to 2015 (CC = 5047), comprising 55 out of 100 articles. Among these 55 papers, 11 articles were published in 2007 (CC = 813), 9 in 2011 (CC = 695), 7 in 2006 (CC = 984), 6 in 2009 (CC = 386), and 5 each in 2008 (CC = 409) and 2010 (CC = 783). The second‐highest number of highly cited articles was released from 1996 to 2005 (CC = 3474), accounting for 32 out of 100, followed by 1986 to 1995 (CC = 1400), with 9 out of 100 articles. However, from 2016 to 2023 (CC = 395), only 4 articles out of 100 were classified as highly cited (Figure 3).

### 3.4 Top Contributing Journals

**Figure 4:**
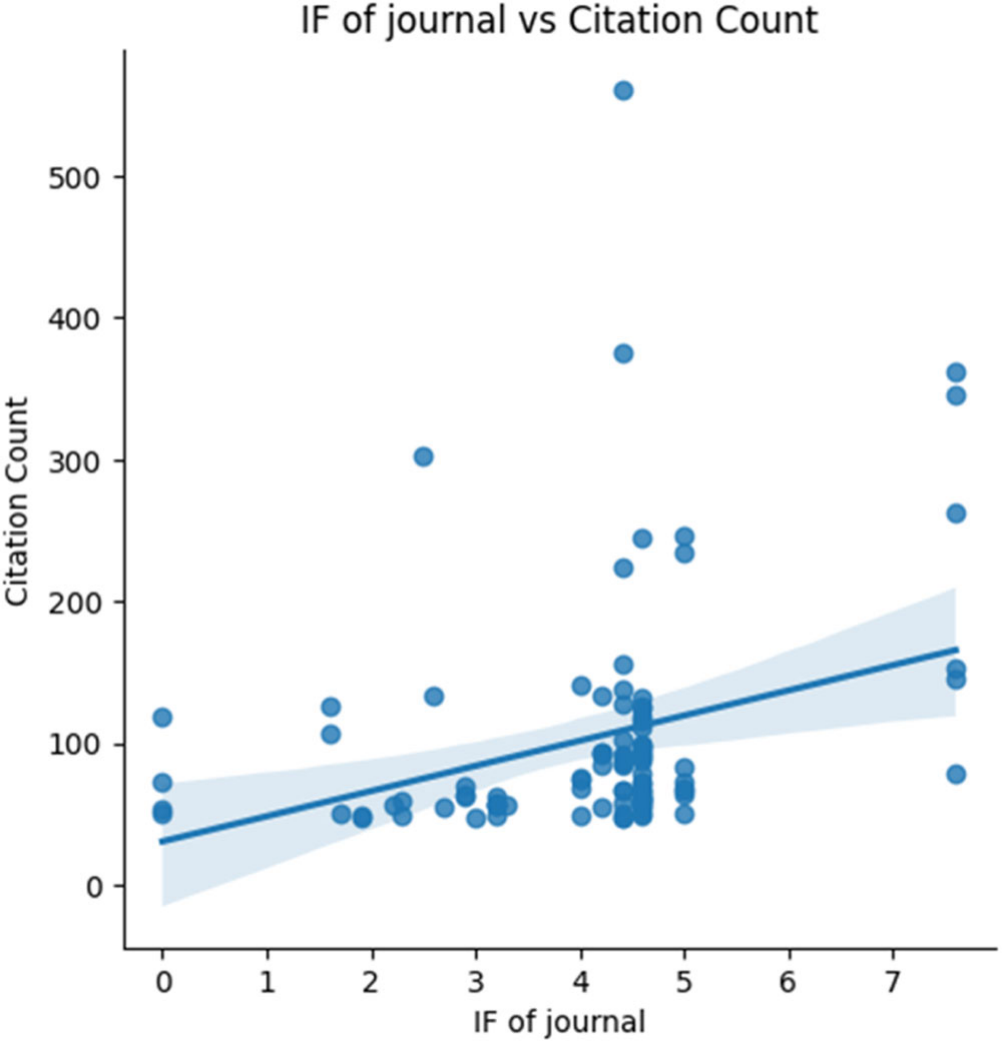
Correlation analysis between the impact factor of a journal and citation count.

The 100 most highly cited selected articles were published across a total of 21 journals, all of which are indexed in Clarivate and possess Journal Impact Factors (JIF) (Table 2). Among these, 7 were classified as JIF Quartile 1 (Q1), 5 were Q3, while 4 journals each fell into Q4 and Q2. Based on the gathered CC, 7 journals ranked as top performers. Among them “*The Journal of Prosthetic Dentistry*” claimed the top position (CC = 2741, *n* = 31), closely trailed by the “*Journal of Dentistry*” (CC = 2460, *n* = 18). The “*Journal of Dental Research*” secured the third position (CC = 1348, *n* = 6), while “Dental Materials” followed closely behind (CC = 1003, *n* = 9). The number of self‐citations ranged from 0 to 48, where *The Journal of Prosthetic Dentistry* ranked as the highest self‐cited journal (48), followed by the *Journal of Esthetic & Restorative Dentistry* (27). When Spearman's correlation coefficient was applied to the CC and the JIF, a statistically significant moderate positive correlation coefficient was found (*r* = 0.35, *p* < 0.05) (Figure 4). This suggests the likelihood that journals with higher IFs tend to receive more citations from other authors compared to those with lower IFs.

**Table 2:** Contributing journals and their metrics in the methods of shade‐matching in dentistry.

Rank	Name of the journal	Journal citation report (JCR) impact factor by clarivate, 2022	Journal impact factor quartile	Number of articles	Number of citations	Number of self‐citations
1.	*Journal of Prosthetic Dentistry*	4.6	Q1	31	2714	48
2.	*Journal of Dentistry*	4.4	Q1	18	2460	22
3.	*Journal of Dental Research*	7.6	Q1	6	1348	20
4.	*Dental Materials*	5.0	Q1	9	1003	4
5.	*Journal of Endodontics*	4.2	Q1	6	552	0
6.	*Journal of Prosthodontics*	4.0	Q1	5	407	14
7.	*Journal of Esthetic and Restorative Dentistry*	3.2	Q1	6	340	27
8.	*Dental Materials Journal*	2.5	Q3	1	303	0
9.	*International Journal of Periodontics and Restorative Dentistry*	1.6	Q4	2	233	1
10.	*Journal of Oral Rehabilitation*	2.9	Q2	3	197	2
11.	*British Dental Journal*	2.6	Q3	1	134	0
12.	*Journal of American Dental Association*	3.9	Not available	2	124	0
13.	*International Journal of Prosthodontics*	2.3	Q3	2	109	6
14.	*International Dental Journal*	3.3	Q2	1	57	1
15.	*Operative Dentistry*	2.2	Q3	1	56	5
16.	*Journal of Applied Oral Science*	2.7	Q2	1	55	0
17.	*International Journal of Advanced Prosthodontics*	2.6	Q3	1	54	1
18.	*International Journal of Computerized Dentistry*	1.7	Q4	1	50	0
19.	*Quintessence International*	1.9	Q4	1	49	0
20.	*American Journal of Orthodontics and Dentofacial Orthopedics*	3.0	Q2	1	48	0
21.	*Journal of Oral Science*	1.9	Q4	1	48	1

### 3.5 Top Contributing Countries

**Figure 5:**
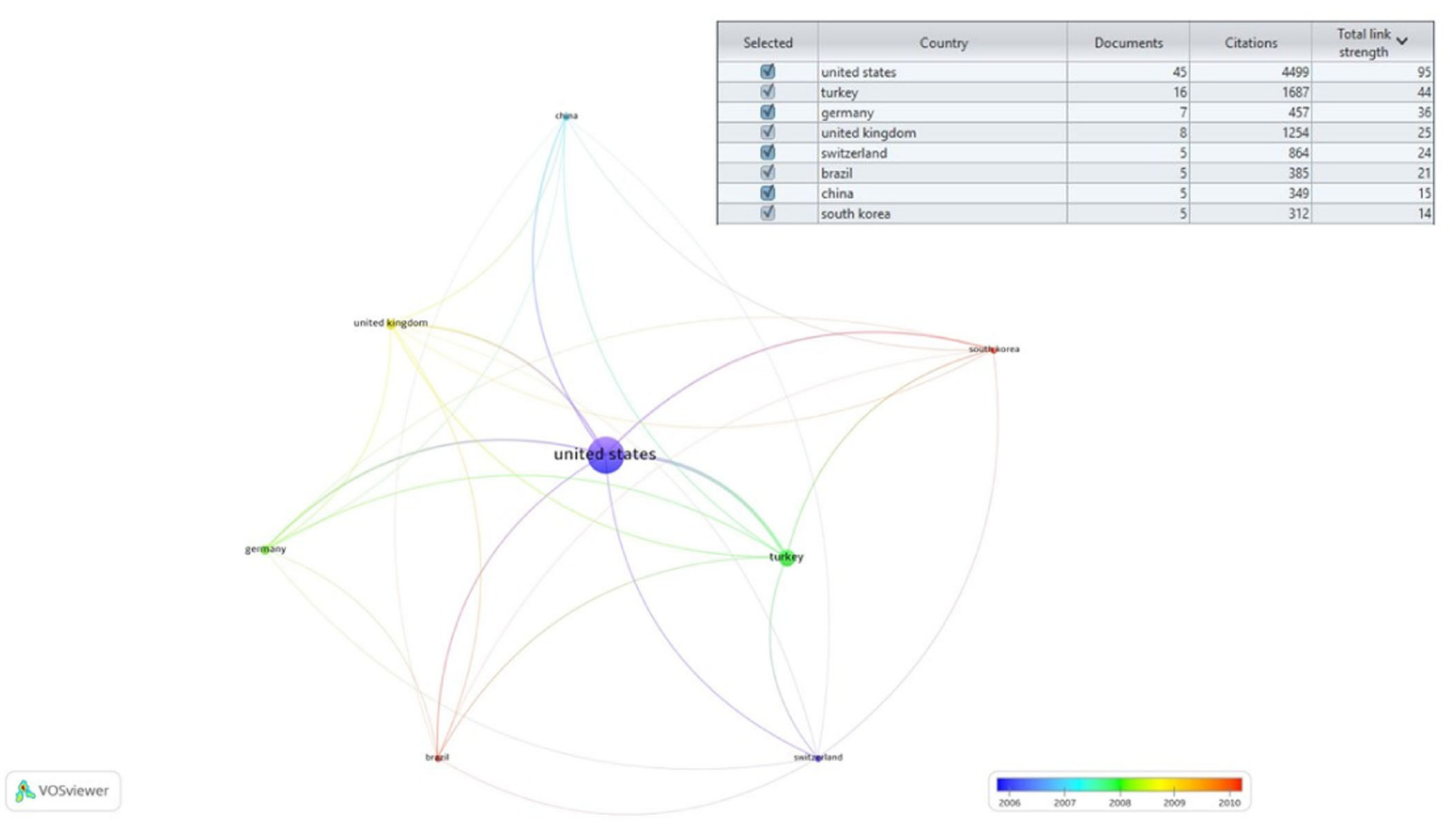
Network analysis of contributing countries in the methods of shade‐analysis.

Among the 28 countries contributing to the method of shade analysis in dentistry, 8 countries met the standard criterion in *VOSviewer* of having at least 5 publications and were subsequently selected for the network analysis. The United States emerged as the leading contributor to the method of color sciences in dentistry (CC = 4499, *n* = 45, average citations per year = 99.98), followed by Turkey (CC = 1687, *n* = 16, average citations per year = 105.44) and then the United Kingdom (CC = 1254, *n* = 8, average citations per year = 156.75). Additionally, Germany, Switzerland, Brazil, China, and South Korea also demonstrated notable contributions in this field (Figure 5).

### 3.6 Top Utilized Keywords

**Figure 6:**
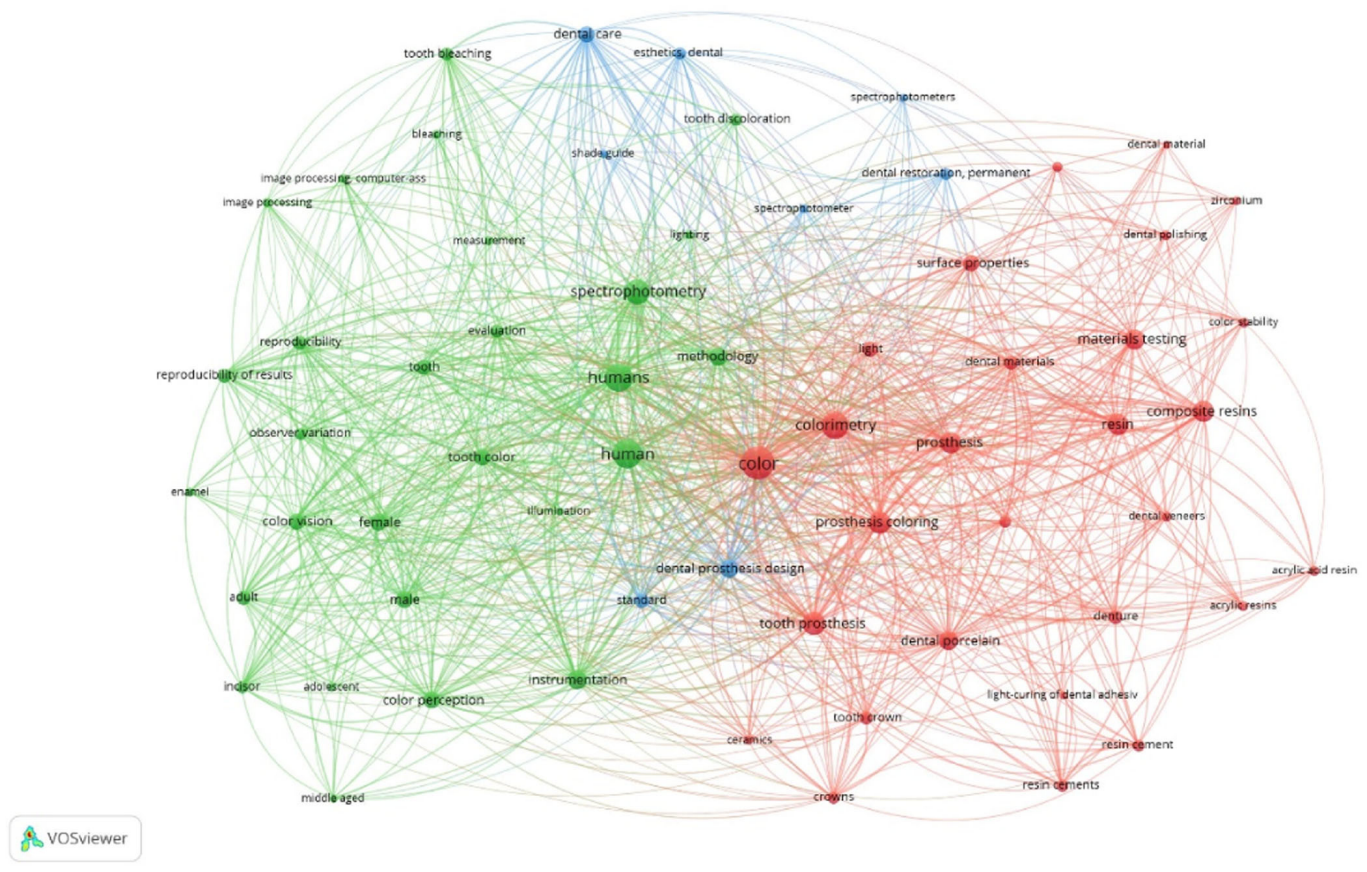
Network analysis between keywords used in the shade‐matching studies.

From 763 keywords provided by the authors, 64 keywords were identified to have appeared at least 5 times, meeting the threshold for inclusion in the *VOSviewer* network analysis. As demonstrated in Figure 6, these keywords were classified into 3 distinct clusters: Cluster 1 (Red points) comprised keywords associated with the color of teeth and restorative materials, Cluster 2 (Green points) is related to human color perception and methods of evaluating colors from tooth surfaces, and Cluster 3 (Blue points) focuses on dental esthetics. Notably, among the selected keywords, “color” emerges as the most frequently used keyword (*n* = 77, total link strength = 841), followed by “human” (*n = 58*, total link strength = 712), and subsequently “colorimetry” (*n* = 50, total link strength = 594) and “spectrophotometry” (*n* *=* 43, total link strength = 478) (Figure 6).

### 3.7 Top Contributing Authors

**Figure 7:**
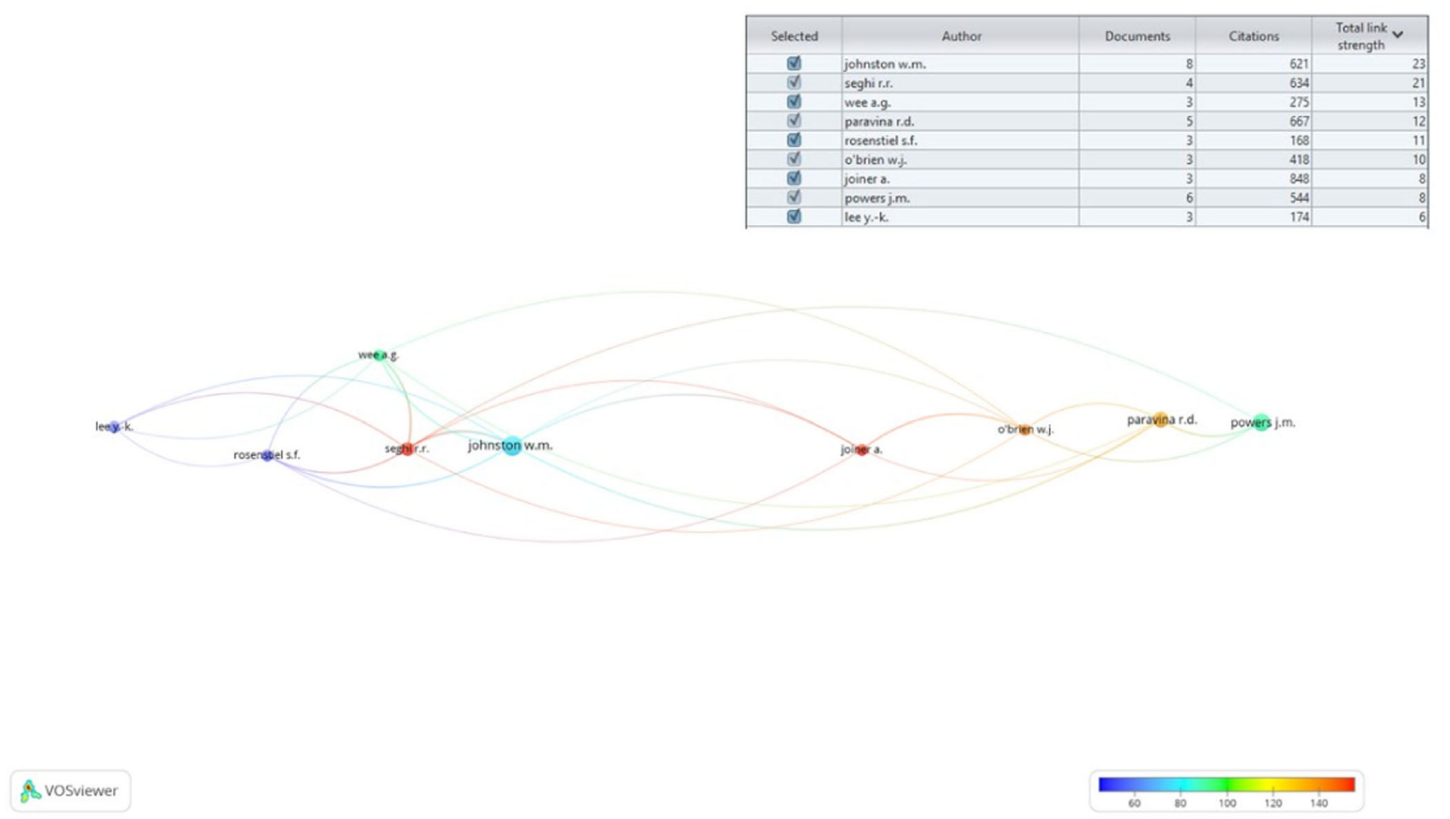
Network analysis of contributing authors in the methods of shade‐analysis.

Among the 311 authors contributing to the shade analysis, only 9 authors surpassed the threshold of having at least 3 published articles, thereby qualifying for network analysis. As shown in Figure 7, the highest number of articles were published by Johnston (*n* = 8, citations per article = 77.62, total link strength = 23), closely followed by Powers (*n* = 6, citations per article = 90.67, total link strength = 8), and Paravina (*n* = 5, citations per article = 133.40, total link strength = 12). The network analysis further highlights the strong network connections that Johnston has with other influential authors, such as Seghi (total link strength = 21), O'Brien (total link strength = 10), and Powers (total link strength = 8). Additionally, a notable connection between Seghi and Powers underscores their significance within the network, indicating their collaborative influence in the field of shade‐matching in dentistry (Figure 7).

### 3.8 Study Design, Nature of Study, Study Samples, and Method of Detecting Shade

Within the 100 articles selected for this bibliometric analysis, experimental studies constituted 60% of the total (CC = 5749), followed by case‐control with 12% (CC = 1005) and review articles with 5% (CC = 1008) (Table 3). Conversely, clinical trials represented only 1% of the selected articles (CC = 48). Regarding the nature of the study, the majority of articles were in‐vitro studies, accounting for 72.83% of the total (*n* = 67, CC = 6379), followed by in‐vivo studies at 16.30% (*n* = 15, CC = 1454), and ex‐vivo studies at 8.70% (*n* = 8, CC = 821). In terms of study samples, restorative materials are the most prevalent, comprising 61.96% of the selected articles (*n* = 57, CC = 5564), followed by studies incorporating tooth samples alongside shade guides (13.04%, *n* = 12, CC = 1074) and tooth samples alone (11.96%, *n* = 11, CC = 1228). The methods used for shade detection varied, with instrumental spectrophotometry being the most frequently employed (42.39%, *n* = 39, CC = 3473), followed by instrumental colorimetry (26.09%, *n* = 23, CC = 2302). Additionally, a significant portion of articles utilized both visual and instrumental methods (26.09%, *n* = 24, CC = 2597), while visual methods alone were the least used (*n* = 3, CC = 262) (Table 3).

**Table 3:** Study design, nature of study, study samples, and method of detecting shade of the 100 most cited articles in the methods of shade‐matching in dentistry.

Study design	Number of articles	Percentage distribution of selected articles (out of 100) (%)	Number of citations
Experimental study	60	60	5749
Case–control study	12	12	1005
Clinical observational study	12	12	1542
Review articles	5	5	1008
Comparative observational study	5	5	338
Conference proceedings	3	3%	489
Observational cross‐sectional study	2	2%	162
Clinical trials	1	1%	48

**Nature of study**	**Number of articles**	**Percentage distribution of selected articles (out of 92)**	**Number of citations**
In‐vitro	67	72.83	6379
In‐vivo	15	16.30	1454
Ex‐vivo	8	8.70	821
Both in‐vivo and in‐vitro	2	2.17	190

**Study samples**	**Number of articles**	**Percentage distribution of selected articles (out of 92)**	**Number of citations**
Restorative material	57	61.96	5564
Tooth along with shade guide	12	13.04	1074
Tooth	11	11.96	1228
Tooth shade guide	5	5.43	492
Tooth along with restorative material	3	3.26	227
Tooth shade guide along with restorative material	3	3.26	167
Computerized samples	1	1.09	92

**Method of detecting shade**	**Number of articles**	**Percentage distribution of selected articles (out of 92)**	**Number of citations**
Instrumental—spectrophotometric	39	42.39	3473
Instrumental—colorimetric	23	26.09	2302
Instrumental—spectroradiometer	2	2.17	146
Instrumental—photographic	1	1.09	64
Both Visual and Instrumental	24	26.09	2597
Visual	3	3.26	262

## 4 Discussion

In the past few decades, authors have used different approaches to address the enigma of dental color matching, driven by the growth of different new generations of technologies dedicated to analyzing, communicating, and verifying shade taking in dentistry (Sadowsky 2006). Acknowledging these scholarly contributions will allow individuals to highlight research as an indicator of progress in this important field. This can be achieved through a concept introduced by Garfield in bibliometric analysis, which involves identifying “Citation Classics,” which are highly cited publications recognized by indices such as the Science Citation Index, the Social Sciences Citation Index, or the Arts and Humanities Citation Index (Praveen et al. 2020). To the best of the author's knowledge, this is the first bibliometric analysis to explore the 100 most cited articles that have contributed to the changing trends in shade‐matching methodologies within dentistry.

One approach to identifying citation classics involves selecting various papers from the top of the list of the top‐cited papers. The present study utilized this approach, employing the “Scopus” database as the primary search engine, which is a comprehensive database covering MEDLINE and EMBASE that streamlines data collection and enhances bibliometric analysis by providing access to authors' profiles and institutional addresses (Kolle et al. 2018; Shome et al. 2023). While alternative influential online databases such as WoS, PubMed, and Google Scholar exist, they often produce varied file formats, potentially leading to human errors. Therefore, to mitigate the risk of any inconsistencies, this study chose to employ a single database to conduct a comprehensive bibliometric analysis (Donthu et al. 2021). Additionally, the search process followed predefined inclusion and exclusion criteria, incorporating Boolean Logic with wildcard characters. This ensured consistency and enabled the integration of related concepts and synonyms, ultimately producing comprehensive results (Aromataris and Riitano 2014).

While performing a bibliometric analysis, “Citation Count” is an important parameter to signify the influence of research work in the given discipline and quantify the number of researchers who contributed to that field. As outlined by Ahmad, Della Bella, Stoddart (2020) and Andersen, Belmont, and Cho (2006), papers with over 100 citations are typically deemed “Classics.” Among the selected 100 most cited articles, 28 received more than 100 citations, ranging from 102 to 560, underscoring the presence of Classic articles in the realm of shade‐matching in dentistry. Additionally, the analysis revealed a total of 10,341 citations for shade‐matching methods in dentistry, which is higher than endodontics (554–87) and orthodontics (545–89) but lower than periodontics (2307–229) and prosthodontics (2368–343) research field (Praveen et al. 2020). One potential explanation for this inconsistency in CC is that citation counts are not standardized and can vary significantly across different fields of study, including within subfields (Bornmann and Daniel 2008).

Among the 100 selected articles, despite articles by Joiner (2004). and Mutlu‐Sagesen et al. (2001) having a 3‐year publication gap, one achieved the top position in terms of citations, while the other ranked lower. This substantial difference in CCs can be explained by the nature of these articles. The article by Jonier is a comprehensive review that delves into fundamental aspects of tooth shade matching, involving topics such as fundamentals of color and color spaces, optical characterization of teeth, color measuring techniques, and individual color perceptions. In contrast, the article by Mutlu‐Sagesen et al. is an experimental study focused on a single instrumental shade analysis method (colorimetry) for detecting the color of restorative material. The broad scope of Jonier's article likely contributes to its high CC, as it serves as a cornerstone resource for researchers and practitioners in the field.

However, our bibliometric analysis highlighted a notable trend, favoring experimental studies over any other studies in the field of shade‐matching in dentistry. Specifically, out of the 100 most cited articles, 60% are experimental studies, and only 1% are clinical trials. One argument supporting this preference is the prevalence of invalid clinical trials in restorative dentistry due to a lack of methodological standardization (Mickenautsch 2020). Thus, authors may opt for experimental studies, which are perceived as a more reliable alternative. Another factor could be the availability and development of study samples. Articles conducting experiments on restorative material samples received more citations (5564 citations) than those conducted on direct tooth surfaces. This suggests a growing interest in characterizing the optical properties of restorative materials, likely driven by ongoing debates surrounding the sustainability of new materials introduced into the industry. Additionally, experimental studies are often less expensive and easier to conduct compared to clinical trials (Shamszadeh, Asgary, and Nosrat 2019), providing researchers with practical reasons to favor them. However, this also indicates a need for clinical evidence‐based research on the topic of shade‐matching. Moreover, the study also reveals a preference for in‐vitro (72.83%) studies over in‐vivo studies (16.30%). One possible justification for this trend is the challenge of standardizing color calibration with in‐vivo settings, where variations in environmental and lighting conditions can significantly increase the chair side color measuring procedure and affect the color measurements (Rashid et al. 2022). Therefore, the authors preferred the in‐vitro nature of the study over in‐vivo studies as it allowed greater control over environmental variables.

Research has suggested that the year of publication may influence an article's CC, with older publications typically receiving more citations than newer ones (Patil et al. 2020). However, both the present bibliometric analysis and the study by Ahmad, Della Bella, and Stoddart (2020) challenged this trend, suggesting that newer publications are cited more frequently than older ones. Our study found the most cited articles were published between 2006 and 2015, while the least cited articles fell within the 1986 to 1995 period. Between 2006 and 2015, online video calling saw a notable surge, fueled by innovations such as camera‐equipped mobile phones (Schnaars and Wymbs 2004), Skype (De Cicco, Mascolo, and Palmisano 2008), and the transition to fourth generation (4G) data connections, enabling high‐quality video calls (Vora 2015). These advancements not only transformed communications but also fueled a growing demand for perfectly matched tooth‐colored restorations among individuals (Rashid, Farook, and Dudley 2023). This phenomenon is also explainable by this bibliometric analysis results, which found a weak positive relationship between CC and age of publication, revealing an increasing relevance of shade matching in both clinical practice and research.

Among the 100 most cited articles, *The Journal of Prosthetic Dentistry* and the *Journal of Dentistry* stood out as the top‐ranked journals, publishing the highest number of articles (31 and 18 articles, respectively) related to shade‐matching in dentistry. This trend is consistent with other bibliometric analyses focusing on dental ceramics and antibacterial dental adhesives (Chen et al. 2021; Khan et al. 2020). The choice of these journals may be attributed to their status as leading international dental journals that exclusively focus on articles in restorative dentistry, aiming to contribute to advancing clinical dental practice. Furthermore, the study reveals that all the top‐ranked journals publishing data on shade matching belong to the first quartile (Q1) with high IFs. The JIF serves as a measure of a journal's prestige and impact in its field. Based on the total citations acquired and correlation analysis between IF and CC, it can be inferred that authors prefer to cite and publish articles related to shade matching in high‐IF journals. This observation aligns with another bibliometric analysis, which found an annual growth rate of 24.3%, in publications related to dentistry in high‐IF journals (Cartes‐Velásquez and Manterola Delgado 2014). Interestingly, when self‐citations were calculated for specific journals (Table 2), *The Journal of Prosthetic Dentistry* and the *Journal of Dentistry* have relatively high self‐citations but still maintain strong JIFs (4.6 and 4.4, respectively), both within the top quartile (Q1). Similarly, the Journal of Dental Research has a higher JIF of 7.6 with 20 self‐citations. These observations suggest that while self‐citations are present, they do not substantially fuel the JIF. This aligns with the findings of Livas and Delli (2018), who noted that while self‐citations might increase the visibility of articles, they do not significantly impact the JIF, thereby reinforcing the influence of these journals beyond their self‐citation practices.

The United States, Turkey, and the United Kingdom emerged as the top‐ranked countries in this bibliometric analysis for their contributions to the method of shade‐matching in dentistry. A study revealed that 34% of adult patients in the United States and 28% in the United Kingdom expressed dissatisfaction with their tooth color (Joiner 2004), indicating a need to develop various methods of color analysis to address treatment related to matching tooth color. Furthermore, the high citation rate of articles from the United States can be attributed to its large scientific community, significant financial resources, and a tendency among authors to cite articles from their own home country (Ahmad, Della Bella, and Stoddart 2020). Another potential reason for the higher citation rates is the significant contribution of authors such as Johnston, Powers, and Paravina, all from the United States, who emerge as the top‐ranked contributors in this bibliometric analysis. These three authors have played pivotal roles in evaluating color science methods in dental research for the past 30 years, placing them at the forefront of the field of dentistry. The network analysis (Figure 7) further revealed a strong collaborative network among these key United States‐based authors, including connections between Johnston, Powers, and Paravina, as well as other influential authors such as Seghi and O'Brien. This interconnection suggests that certain author groups may engage in frequent citation of each other's work, which could imply self‐citation practices within these clusters. While previous studies (Livas and Delli 2018) have noted a decrease in self‐citations among dental journals since 2016, direct evidence of self‐citation specifically within this subfield of dentistry would require further investigation in future studies. While the prominence of these key contributors is evident, it is also important to acknowledge the collaborative efforts within the field of shade‐matching in dentistry. Among the 100 selected articles, many studies involve co‐authors from different countries (Villalta et al. 2006; Vichi, Ferrari, and Davidson 2004; Marciano et al. 2014; Dozić et al. 2007; Joiner et al. 2008), reflecting a degree of international collaboration. However, there is always room for further enhancing international partnerships to enrich the research landscape. Future efforts could focus on fostering more cross‐border collaborations, leveraging diverse perspectives, and combining resources to advance the field of shade‐matching in dentistry.

Keywords play a critical role in research papers, serving as codes to efficiently locate relevant scientific articles. The findings from *VOSviewer* (Figure 6) revealed that the keywords utilized in this analysis are closely interconnected, underscoring the topic's significance. Thus, researchers can utilize these frequently used keywords to locate published articles in the field of color science in dentistry. According to this study's findings, the most frequently used keyword is “color,” reflecting authors' interest and eagerness to explore the topic. Other commonly used keywords include “spectrophotometry” and “colorimetry,” indicating a trend toward instrumental color evaluation in dentistry. Notably, this study also found that nearly 70% of newly published articles favored instrumental over visual methods for detecting color values. This reflects a shift away from older publications that used visual methods for shade analysis. This preference could be attributed to the potential observational biases associated with visual analysis (Rashid, Farook, and Dudley 2023). Among the color measuring instruments, approximately 49% of authors preferred using a spectrophotometer, while 26% opted for a colorimeter, likely due to their reliability in providing accurate color values. Another potential explanation may be that some authors regard these instruments as the “gold standard” (Paul et al. 2002; Heydecke, Zhang, and Razzoog 2001). However, the concept of a fixed “gold standard” is questionable because knowledge is constantly evolving, rendering the term restrictive (Rashid, Farook, and Dudley 2023).

While our bibliometric analysis provided valuable insights into the landscape of shade‐matching methods in dentistry through rigorous data collection and analysis, several limitations must be acknowledged. First, the analysis relied solely on data from one database, albeit a widely used and reliable database. Future studies may use this data as a baseline and consider incorporating additional databases to determine whether there are different results. Second, the study was restricted to English‐language literature and studies that evaluated shade on tooth and restorative material surfaces. This limitation can open some scope for further studies to explore shade‐matching methodologies in non‐English literature and investigate other aspects of dental esthetics beyond tooth and restorative material surfaces. Despite efforts to create comprehensive search strings, it was possible that some relevant articles were missed due to the complexity of the topic and variations in terminology used across publications. Furthermore, the study employed predefined inclusion and exclusion criteria, including a minimum CC of five, to select articles for analysis. While this criterion helped identify influential papers on this specific topic, it may have excluded recently published articles that have not yet accrued significant citations.

## 5 Conclusion

Based on the present study findings, there was a comparatively high CC of published articles for the topic shade‐matching in dentistry, indicating the availability of high‐quality scientific research in this domain. Over the study period, there was a noticeable upward trajectory of CC in shade‐matching in dentistry. Among the various shade analysis methods in dentistry, instrumental analysis, specifically spectrophotometer, emerged as the most frequently employed method over visual analysis. The high prevalence of experimental study designs in the published research suggested a strong need for evidence‐based approaches in addressing the complexities of shade analysis in dentistry. Furthermore, high‐IF journals are identified as influential platforms for showcasing research findings, and the global collaborative nature of shade‐matching research emphasizes the crucial role of international cooperation in advancing dental color science.

## Author Contributions

**James Dudley:** conceptualization, validation, data curation, reviewing and editing, supervision. **Farah Rashid:** conceptualization, methodology, formal analysis, data curation, writing–original draft, editing and project administration.

## Conflicts of Interest

The authors declare no conflicts of interest.

## Supporting information

Supporting information.

Supporting information.

## Data Availability

The data is accessible in Tables and Supporting Information files. Additional data can be provided upon request.
