# Supplementary material for: Bibliometric Analysis of the 100 Most‐Cited Articles on the Methods of Shade‐Matching in Dentistry
Source: Clin Exp Dent Res. 2024 Nov 3;10(6):e70037. doi: 10.1002/cre2.70037 (PMC11532372; doi:10.1002/cre2.70037)
Supplement: Supplementary file 1 — Supporting information. [file CRE2-10-e70037-s001.docx]

**Supplementary Table 1**: Articles excluded from this bibliometric analysis along with the reasons for their exclusion

| **Number** | **Year** | **Title** | **Reasons for exclusion** |
| --- | --- | --- | --- |
|  | 1976 | An infrared spectroscopic study of sealants | No shade analysis was done |
|  | 1985 | Systematic Assessment of Color Removal Following Vital Bleaching of Intrinsically Stained Teeth | The method of detecting the colors is unclear in the abstract |
|  | 1986 | Spectrophotometric analysis of color differences between porcelain systems | Abstract is not available |
|  | 1991 | A spectrophotometric analysis of dentinal leakage in the resected root | No shade analysis was done |
|  | 1993 | Evaluating tooth color change from carbamide peroxide gel | The method of detecting the colors is unclear in the abstract |
|  | 1994 | The sensitivity and specificity of a colorimetric microbiological caries activity test (Cariostat) in preschool children | No shade analysis was done |
|  | 1995 | Effect of bleaching on microhardness, morphology, and color of enamel | Abstract is not available |
|  | 1996 | Spectroscopic changes in human dentine exposed to various storage solutions - Short term | No shade analysis was done |
|  | 1998 | Parents' satisfaction with children's tooth color: Fluorosis as a contributing factor | Survey |
|  | 2001 | Influence of Posts and Cores on Light Transmission Through Different All-Ceramic Crowns: Spectrophotometric and Clinical Evaluation | No shade analysis was done |
|  | 2002 | Relationship between tooth shade value and skin color: An observational study | Falls under one of the exclusion criteria of this study (refer to exclusion criteria 3) |
|  | 2003 | Effects of in-office bleaching products on surface finish of tooth-colored restorations | No shade analysis was done |
|  | 2004 | Prevalence of self-assessed tooth discolouration in the United Kingdom | Survey |
|  | 2004 | Extraradicular diffusion of hydrogen peroxide and pH changes associated with intracoronal bleaching of discoloured teeth using different bleaching agents | No shade analysis was done |
|  | 2004 | In vitro fracture resistance and marginal adaptation of metallic and tooth-coloured post systems | No shade analysis was done |
|  | 2004 | Comparison of in vitro tensile bond strengths of luting cements to metallic and tooth-colored posts | No shade analysis was done |
|  | 2004 | Finishing Tooth-Colored Restorations in Vitro: An Index of Surface Alteration and Finish-line Destruction | Non-human teeth used as study sample |
|  | 2004 | The effect of home bleaching agents on the surface roughness of tooth-colored restoratives with time | No shade analysis was done |
|  | 2004 | Development of methods to enhance extrinsic tooth discoloration for comparison of toothpastes: 2. Two-product clinical study | No shade analysis was done |
|  | 2005 | Age and perception of dental appearance and tooth colour | Survey |
|  | 2005 | Effect of two different bleaching regimens on the gloss of tooth colored restorative materials | No shade analysis was done |
|  | 2006 | In vitro colorimetric evaluation of the efficacy of various bleaching methods and products | Non-human teeth used as study sample |
|  | 2007 | Uv-vis spectrophotometric direct transmittance analysis of composite resins | Study evaluated light transmission percentages of composite resins |
|  | 2007 | Radiometric and spectrophotometric analysis of third generation light-emitting diode (LED) light-curing units | No shade analysis was done |
|  | 2008 | The effect of bleaching, varying the shade or thickness of composite veneers on final colour: An in vitro study | Non-human teeth used as study sample |
|  | 2008 | The influence of tooth colour on the perceptions of personal characteristics among female dental patients: Comparisons of unmodified, decayed and 'whitened' teeth | Survey |
|  | 2008 | The effect of debonding and/or bleaching on enamel color - An in-vitro study | Non-human teeth used as study sample |
|  | 2009 | The effect of coffee solution on tooth color during home bleaching applications | Non-human teeth used as study sample |
|  | 2009 | Effect of light irradiation on tooth whitening: Enamel microhardness and color change | Non-human teeth used as study sample |
|  | 2009 | Indirect resin composite restorations: evaluation of polymerization of luting agents by means of micro-Raman spectrophotometry | No shade analysis was done |
|  | 2010 | The evaluation of a novel method comparing quantitative light-induced fluorescence (QLF) with spectrophotometry to assess staining and bleaching of teeth | Non-human teeth used as study sample |
|  | 2010 | An investigation into the effect of try-in pastes, uncured and cured resin cements on the overall color of ceramic veneer restorations: An in vitro study | Non-human teeth used as study sample |
|  | 2010 | Teaching of color in predoctoral and postdoctoral dental education in 2009 | Survey |
|  | 2012 | Randomized clinical trial of the efficacy, tolerability, and long-term color stability of two bleaching techniques: 18-month follow-up | Tooth shade evaluated from microstructural level by using Scanning electron microscopy (SEM) technique |
|  | 2012 | The effect of at-home bleaching and toothbrushing on removal of coffee and cigarette smoke stains and color stability of enamel | Non-human teeth used as study sample |
|  | 2013 | Chromatic analysis of teeth exposed to different mouthrinses | Non-human teeth used as study sample |
|  | 2013 | Bond strength of different resin cement and ceramic shades bonded to dentin | No shade analysis was done |
|  | 2014 | Spectrophotometric analysis of crown discoloration induced by various antibiotic pastes used in revascularization | Non-human teeth used as study sample |
|  | 2014 | Effect of cleanser solutions on the color of acrylic resins associated with titanium and nickel-chromium alloys | Performed shade analysis on metal alloys |
|  | 2014 | Spectrophotometric evaluation of dental bleaching under orthodontic bracket in enamel and dentin | Non-human teeth used as study sample |
|  | 2014 | Assessment of color stability of white mineral trioxide aggregate angelus and bismuth oxide in contact with tooth structure | Non-human teeth used as study sample |
|  | 2014 | Color stability and polymerization behavior of direct esthetic restorations | Non-human teeth used as study sample |
|  | 2014 | Color stability of white mineral trioxide aggregate in contact with hypochlorite solution | Tooth shade evaluated from microstructural level by using Fourier transform infrared spectroscopy technique |
|  | 2015 | Influence of staining solution and bleaching on color stability of resin used for caries infiltration | Non-human teeth used as study sample |
|  | 2015 | Peri-implant soft tissue colour around titanium and zirconia abutments: A prospective randomized controlled clinical study | Performed shade analysis for peri-implant soft tissue and gingiva |
|  | 2015 | At-home bleaching: Color alteration, hydrogen peroxide diffusion and cytotoxicity | Non-human teeth used as study sample |
|  | 2015 | Apical extrusion of sodium hypochlorite activated with two laser systems and ultrasonics: A spectrophotometric analysis | No shade analysis was done |
|  | 2015 | A novel colourimetric technique to assess chewing function using two-coloured specimens: Validation and application | No shade analysis was done |
|  | 2015 | Comparison of bleaching efficacy of two bleaching agents on teeth discoloured by different antibiotic combinations used in revascularization | Non-human teeth used as study sample |
|  | 2016 | An in situ study of the influence of staining beverages on color alteration of bleached teeth | Non-human teeth used as study sample |
|  | 2016 | Abutment Material Effect on Peri-implant Soft Tissue Color and Perceived Esthetics | Performed shade analysis on peri-implant soft tissue |
|  | 2016 | Structural color changes in permanent enamel of patients with cleft lip and palate: a case–control study | No shade analysis was done |
|  | 2016 | Influence of enamel thickness on bleaching efficacy: An in-depth color analysis | Non-human teeth used as study sample |
|  | 2016 | Clinical and spectrophotometric evaluation after Chlorhexidine use in periodontal flap surgery: A prospective randomized clinical trial | The method of detecting the colors is unclear in the abstract |
|  | 2017 | Enamel mineral content changes after bleaching with high and low hydrogen peroxide concentrations: Colorimetric spectrophotometry and total reflection X-ray fluorescence analyses | No shade analysis was done |
|  | 2017 | Crown discoloration promoted by materials used in regenerative endodontic procedures and effect of dental bleaching: Spectrophotometric analysis | Non-human teeth used as study sample |
|  | 2017 | Spectrophotometric analysis of fluorescent zirconia abutments compared to “conventional” zirconia abutments: A within subject controlled clinical trial | Performed shade analysis on gingiva |
|  | 2018 | Optimized approach of dental composites identification with the use of original spectrophotometric algorithm | No shade analysis was done |
|  | 2019 | Influence of the composition and shades of ceramics on light transmission and degree of conversion of dual-cured resin cements | No shade analysis was done |
|  | 2019 | A systematic review and meta-analysis of the influence of abutment material on peri-implant soft tissue color measured using spectrophotometry | Shade analysis was evaluated on peri-implant soft tissue |
|  | 2019 | Color stability of ceramic veneers cemented with self-adhesive cements after accelerated aging | Non-human teeth used as study sample |
|  | 2020 | The relationship of tooth shade and skin tone and its influence on the smile attractiveness | Falls under one of the exclusion criteria of this study (refer to exclusion criteria 3) |
|  | 2020 | Spectrophotometric analysis evaluating apical microleakage in retrograde filling using GIC, MTA and biodentine: An in-vitro study | No shade analysis was done |
|  | 2020 | A laboratory evaluation of cell viability, radiopacity and tooth discoloration induced by regenerative endodontic materials | Non-human teeth used as study sample |
|  | 2020 | Color stability of Bulk-Fill composite restorations | Non-human teeth used as study sample |
|  | 2021 | Biological parameters, discolouration and radiopacity of calcium silicate-based materials in a simulated model of partial pulpotomy | Non-human teeth used as study sample |
