# Supplementary material for: Bibliometric Analysis of the 100 Most‐Cited Articles on the Methods of Shade‐Matching in Dentistry
Source: Clin Exp Dent Res. 2024 Nov 3;10(6):e70037. doi: 10.1002/cre2.70037 (PMC11532372; doi:10.1002/cre2.70037)
Supplement: Supplementary file 2 — Supporting information. [file CRE2-10-e70037-s002.docx]

**Supplementary Table 2**: Python codes script for statistical analysis

| **Code Serial** | **Codes** |
| --- | --- |
| 1 | import pandas as pd  import numpy as np  import seaborn as sns  import matplotlib.pyplot as plt |
| 2 | data = pd.read_csv("Citation analysis data.csv") |
| 3 | from scipy.stats import shapiro  from statsmodels.graphics.gofplots import qqplot  import matplotlib.pyplot as plt  # Shapiro-Wilk Test for normality  stat, p = shapiro(data['Cites'])  print("Shapiro-Wilk Test - p-value:", p)  if p > 0.05:  print("Data appears to be normally distributed")  else:  print("Data does not appear to be normally distributed") |
| 4 | from scipy.stats import shapiro  from statsmodels.graphics.gofplots import qqplot  import matplotlib.pyplot as plt  # Shapiro-Wilk Test for normality  stat, p = shapiro(data['Age of publication'])  print("Shapiro-Wilk Test - p-value:", p)  if p > 0.05:  print("Data appears to be normally distributed")  else:  print("Data does not appear to be normally distributed") |
| 5 | from scipy.stats import shapiro  from statsmodels.graphics.gofplots import qqplot  import matplotlib.pyplot as plt  # Shapiro-Wilk Test for normality  stat, p = shapiro(data['Density'])  print("Shapiro-Wilk Test - p-value:", p)  if p > 0.05:  print("Data appears to be normally distributed")  else:  print("Data does not appear to be normally distributed") |
| 6 | from scipy.stats import shapiro  from statsmodels.graphics.gofplots import qqplot  import matplotlib.pyplot as plt  # Shapiro-Wilk Test for normality  stat, p = shapiro(data['IF of journal'])  print("Shapiro-Wilk Test - p-value:", p)  if p > 0.05:  print("Data appears to be normally distributed")  else:  print("Data does not appear to be normally distributed") |
| 7 | from scipy.stats import spearmanr  # Calculate Spearman's correlation coefficient if data is not normally distributed  spearman_corr_coef, spearman_p_value = spearmanr(data['Cites'], data['Age of publication'])  print("Spearman's correlation coefficient:", spearman_corr_coef)  print("p-value:", spearman_p_value) |
| 8 | # Scatter plot with regression line  sns.lmplot(x='Age of publication', y='Cites', data=data)  plt.title("Citation Count vs Age of Publication")  plt.xlabel("Age of Publication")  plt.ylabel("Citation Count")  plt.show() |
| 9 | from scipy.stats import spearmanr  # Calculate Spearman's correlation coefficient if data is not normally distributed  spearman_corr_coef, spearman_p_value = spearmanr(data['Density'], data['Age of publication'])  print("Spearman's correlation coefficient:", spearman_corr_coef)  print("p-value:", spearman_p_value) |
| 10 | # Scatter plot with regression line  sns.lmplot(x='Age of publication', y='Density', data=data)  plt.title("Density vs Age of Publication")  plt.xlabel("Age of Publication")  plt.ylabel("Density")  plt.show() |
| 11 | from scipy.stats import spearmanr  # Calculate Spearman's correlation coefficient if data is not normally distributed  spearman_corr_coef, spearman_p_value = spearmanr(data['Cites'], data['IF of journal'])  print("Spearman's correlation coefficient:", spearman_corr_coef)  print("p-value:", spearman_p_value) |
| 12 | # Scatter plot with regression line  sns.lmplot(x='IF of journal', y='Cites', data=data)  plt.title("IF of journal vs Citation Count")  plt.xlabel("IF of journal")  plt.ylabel("Citation Count")  plt.show() |
